# Molecular Mechanisms of L1 and NCAM Adhesion Molecules in Synaptic Pruning, Plasticity, and Stabilization

**DOI:** 10.3389/fcell.2021.625340

**Published:** 2021-01-28

**Authors:** Bryce W. Duncan, Kelsey E. Murphy, Patricia F. Maness

**Affiliations:** Department of Biochemistry and Biophysics, Neuroscience Research Center, Carolina Institute for Developmental Disabilities, University of North Carolina School of Medicine, Chapel Hill, NC, United States

**Keywords:** synapse, cell adhesion molecule, ankyrin, perineuronal net, synaptic stabilization

## Abstract

Mammalian brain circuits are wired by dynamic formation and remodeling during development to produce a balance of excitatory and inhibitory synapses. Synaptic regulation is mediated by a complex network of proteins including immunoglobulin (Ig)- class cell adhesion molecules (CAMs), structural and signal-transducing components at the pre- and post-synaptic membranes, and the extracellular protein matrix. This review explores the current understanding of developmental synapse regulation mediated by L1 and NCAM family CAMs. Excitatory and inhibitory synapses undergo formation and remodeling through neuronal CAMs and receptor-ligand interactions. These responses result in pruning inactive dendritic spines and perisomatic contacts, or synaptic strengthening during critical periods of plasticity. Ankyrins engage neural adhesion molecules of the L1 family (L1-CAMs) to promote synaptic stability. Chondroitin sulfates, hyaluronic acid, tenascin-R, and linker proteins comprising the perineuronal net interact with L1-CAMs and NCAM, stabilizing synaptic contacts and limiting plasticity as critical periods close. Understanding neuronal adhesion signaling and synaptic targeting provides insight into normal development as well as synaptic connectivity disorders including autism, schizophrenia, and intellectual disability.

## Introduction

Formation of synaptic contacts, pruning of axonal and dendritic processes, and elimination of synapses occurs during development of the mammalian brain and is vital for establishment of neuronal circuitry. These dynamic responses occur within both excitatory and inhibitory neurons in early postnatal life, are often associated with critical periods of plasticity, and can be dependent on neural activity. It is thought that overproduction of neuronal processes and nascent synapses followed by selective pruning, serves to stabilize active connections and eliminate less active ones, resulting in an appropriate excitatory-inhibitory balance in cortical networks. Identification of postnatal mechanisms for regulating synapse density is important for understanding how normal circuits are formed, as well as providing insight into how these circuits may be altered in neurodevelopmental disorders such as autism, schizophrenia, bipolar disorder and intellectual disability (Forrest et al., 2018).

Defective pruning of dendritic spines and excitatory synapses in the human brain is a leading hypothesis to explain increased spine density in frontal cortical areas, and social and cognitive impairments seen in autism and Fragile X syndrome (Hutsler and Zhang, 2010; Tang et al., 2014). Excessive spine pruning in the prefrontal cortex may conversely contribute to decreased spine density in schizophrenia and bipolar disorder (Konopaske et al., 2014; Phillips and Pozzo-Miller, 2015). Recent evidence has revealed that inhibitory cortical connections display significant levels of remodeling during the juvenile to adult transition. Among the numerous types of interneurons, parvalbumin (PV)-expressing basket cells display active remodeling in postnatal development at the perisomatic region of cortical pyramidal cells (Sullivan et al., 2020). These interneurons regulate synchronous firing, gamma rhythms, and working memory, all of which are impaired in schizophrenia (Gonzalez-Burgos et al., 2015).

Immunoglobulin (Ig) class cell adhesion molecules are transmembrane glycoproteins that are widely expressed in the mammalian nervous stem where they regulate diverse aspects of brain development. L1-CAMs, NCAM, as well as Synaptic Cell Adhesion Molecules (SynCAM1-4) are among the most well-studied regulators of synaptic development and plasticity. This review focuses on the role of this group of adhesion molecules in synapse remodeling and stabilization of excitatory and inhibitory connections during maturation of neural circuits. Other recent reviews cover the role of IgCAMs in synapse formation (Sytnyk et al., 2017; Cameron and McAllister, 2018).

## Dendritic Spine Pruning Through IgCAMs and Semaphorins

Neuronal circuits in the brain comprise two main types of neurons: excitatory pyramidal neurons, which use glutamate for neurotransmission, and inhibitory interneurons, which use gamma amino butyric acid (GABA). Pyramidal cells constitute approximately 80–90% of the neuronal population, whereas interneurons comprise 10–20% (Hu et al., 2014). Most excitatory synapses in the mammalian brain form on spines, small protrusions from pyramidal cell dendrites (Yuste et al., 2000). During development and adulthood, dendritic spines are subject to controlled elimination (pruning), which refines neural circuits and establishes appropriate excitatory-inhibitory balance. Activity-dependent spine elimination has been extensively studied in the adult brain (Stein and Zito, 2019), however, less is known about earlier postnatal stages of spine pruning. L1-CAMs are established promoters of neural adhesion, migration, and process outgrowth (Maness and Schachner, 2007). Among these, NrCAM and CHL1 were found to mediate adolescent spine pruning in mouse genetic models (Demyanenko et al., 2014; Mohan et al., 2019a,b). Variations in the NrCAM gene have been implicated in autism (Hutcheson et al., 2004; Bonora et al., 2005; Sakurai et al., 2006; Marui et al., 2009; Pinto et al., 2010; Voineagu et al., 2011) and schizophrenia (Kim et al., 2009; Ayalew et al., 2012; Zhang et al., 2015), while mutations in the CHL1 (CALL) gene are linked to intellectual disability (Angeloni et al., 1999; Frints et al., 2003; Cuoco et al., 2011), schizophrenia (Sakurai et al., 2002; Chen et al., 2005; Chu and Liu, 2010), and autism (Salyakina et al., 2011).

**FIGURE 1:**
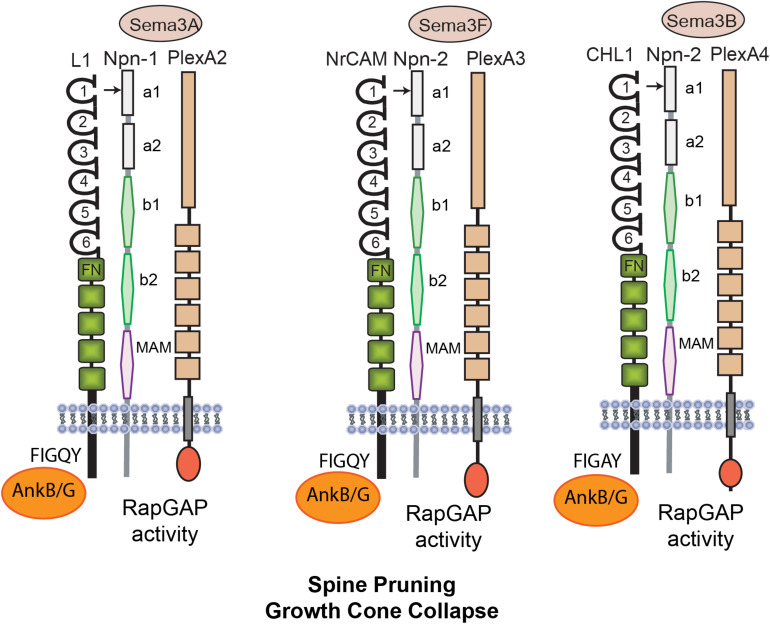
Mechanisms of L1 Family Mediated Spine Pruning. L1 is a transmembrane glycoprotein with 6 Ig and 5 FNIII domains and a short cytoplasmic tail. Homophilic binding in *cis* and *trans* is mediated principally by the Ig2 domain, which is present within a folded horseshoe conformation of Ig1-4. The L1 Ig1 domain binds heterophilically to the Sema3A co-receptor Neuropilin-1 (Npn1). Npn1/2 consist of 2 CUB domains (a1, a2), 2 coagulation factor V/VII domains (b1, b2) and a meprin-A5-mu domain (MAM). Other L1-CAMs Close Homolog of L1 (CHL1) and NrCAM have similar structures and carry out related functions. The NrCAM Ig1 domain constitutively binds Npn2 at its a1 domain and mediates responses to Sema3F in complex with PlexA3. Activation of the intrinsic Rap-GAP activity of PlexAs downregulates Rap1-GTPase. CHL1 binds Npn2 and mediates similar responses to Sema3B in complex with PlexA4. All L1-CAMs reversibly bind AnkyrinB/G (AnkB/G), a spectrin-actin adaptor protein, at a conserved motif FIGQ/AY in the cytoplasmic domain.

As demonstrated in mouse model systems L1-CAMs induce neurite outgrowth through homophilic binding, and they also promote axon repulsion and growth cone collapse in response to secreted class 3 Semaphorins (Sema3) (Castellani et al., 2000; Wright et al., 2007; Bechara et al., 2008; Mohan et al., 2019a). Interestingly, two of these repellent ligands, Sema3F and Sema3B, induce retraction and loss of dendritic spines in postnatally developing cortical pyramidal neurons (Tran et al., 2009; Demyanenko et al., 2014; Mohan et al., 2019b). These ligands display striking selectivity in pruning different populations of spines, even on the same dendrite. Such selective spine pruning is achieved through a combinatorial mechanism in which Sema3 holoreceptor complexes comprising L1-CAMs, Neuropilins (Npn1/2), and PlexinA subunits (PlexA1-4) associate in different combinations to transduce intracellular signals leading to spine collapse (Figure 1). Both NrCAM and CHL1 constitutively bind Npn2, which in turn engages different PlexA subunits and associated L1-CAM molecules (Demyanenko et al., 2014; Mohan et al., 2019a,b). The NrCAM, Npn2, PlexA3 complex mediates Sema3F-induced spine pruning (Demyanenko et al., 2014), whereas the CHL1, Npn2, PlexA4 complex mediates Sema3B-induced spine pruning (Mohan et al., 2019b). Neuropilins bind PlexAs with modest affinity, however, affinity is increased upon binding of dimerized Sema3 ligands (Janssen et al., 2012). Sema5A, a transmembrane Semaphorin, also regulates dendritic spines by signaling through PlexA2 to suppress spinogenesis and inhibit excitatory synapse formation in dentate gyrus granule cells (Duan et al., 2014).

Single null mutant mice lacking NrCAM, CHL1, Npn2, PlexA3, or Sema3F display elevated spine and excitatory synapse density as shown in several neocortical areas (Tran et al., 2007; Demyanenko et al., 2014; Mohan et al., 2019b). This phenotype is restricted to apical dendrites, where Npn2 is preferentially localized (Tran et al., 2009). Selective spine pruning on apical dendrites is of interest with regard to cortical network establishment, as apical dendrites differ from basal dendrites in responding more robustly to intra-cortical and thalamo-cortical inputs, and having distinctive temporal plasticity rules (Gordon et al., 2006; Shigematsu et al., 2016). Excitatory neurotransmission is increased in NrCAM and CHL1-deficient prefrontal slices, consistent with cortical hyperexcitability (Demyanenko et al., 2014; Mohan et al., 2019b). It is of interest that Sema3B (Mohan et al., 2019b) and Sema3F (Orr et al., 2017; Wang et al., 2017) are secreted in an activity-dependent manner, as shown in cultures, raising the possibility that limited diffusion of Sema3 ligands from spines may prune less active, immature neighbors to refine cortical circuits. Indeed, spines with immature, thin morphology are selectively pruned by Sema3F and Sema3B (Demyanenko et al., 2014; Mohan et al., 2019b). The Sema3F/Npn2/PlexA3 complex also plays an adult role in cortical neurons, facilitating homeostatic scaling of synaptic strength by interacting with the glutamate receptor GluA1 (Wang et al., 2017).

The molecular mechanism leading to spine loss is best understood for the Sema3F holoreceptor. Binding of NrCAM to Npn2 occurs in *cis* within the dendritic membrane and is mediated by charged residues within two motifs (TARNER and DDK) in the NrCAM Ig1 domain, which interact with specific residues in the Npn2 a1 domain (Mohan et al., 2019a). Sema3F dimers induce membrane clustering of Npn2 and constitutively associated PlexA3, stimulating the intrinsic Rap-GTPase activating protein (GAP) activity of PlexA3 (Pascoe et al., 2015). This in turn downregulates Rap1 preventing activation of Rap-dependent Rap1-GTP-interacting adaptor molecule (RIAM) and Talin from inducing inside-out signaling of β1 integrins (Mohan et al., 2019a).

Since activation of β1 integrins is known to promote spine stabilization (DePoy et al., 2019), integrin inactivation and loss of adhesion to the extracellular matrix likely contributes to spine pruning. Actin cytoskeletal remodeling resulting from these effector pathways serves to collapse the affected spines.

## Neuroplasticity of Inhibitory Synaptic Connections

The Ig-class adhesion molecule Neural Cell adhesion Molecule (NCAM1) has been extensively studied for its pre-and postsynaptic roles in regulating synaptic development, long term potentiation (LTP), learning, and memory in excitatory pyramidal neurons of the hippocampus and cerebral cortex [reviewed in Sytnyk et al. (2017)]. In pyramidal neurons, NCAM induces synaptogenesis (Dityatev et al., 2004) as well as endocytosis of pre-synaptic vesicles (Shetty et al., 2013). Current focus has shifted to the role of NCAM in regulating connectivity of GABAergic interneurons, in particular the targeting of the basket interneuron inputs to the perisomatic region of cortical pyramidal neurons.

GABAergic interneurons function to limit the activity of pyramidal neurons thus regulating firing, timing, and synchrony of cortical circuits (Hu et al., 2014). Interneurons are highly diverse and express different markers: calcium binding proteins [parvalbumin (PV), calbindin, and calretinin] and neuropeptides [cholecystokinin (CCK), somatostatin, and vasoactive intestinal peptide] (Wamsley and Fishell, 2017). Basket cells are PV-expressing interneurons that synaptically target the soma and proximal apical dendrite of pyramidal cells. Perisomatic inhibitory synapses derive chiefly from PV+ basket cells but also from some CCK+ interneurons (Karson et al., 2009; Wyeth et al., 2010; Hansen et al., 2017; Veres et al., 2017; Horn and Nicoll, 2018). Perisomatic inhibition is vital for pyramidal cell synchrony, working memory, decision making, and social behavior (Lagler et al., 2016; Ferguson and Gao, 2018), all of which are impaired in neuropsychiatric diseases (Dienel and Lewis, 2019). PV-expressing chandelier interneurons target the axon initial segment (AIS) of pyramidal neurons, where action potentials are initiated (Somogyi, 1977). The density of inhibitory boutons derived from chandelier interneurons onto the AIS has been reported to be increased in layer 2/3 of the dorsolateral prefrontal cortex in schizophrenia (Rocco et al., 2017).

**FIGURE 2:**
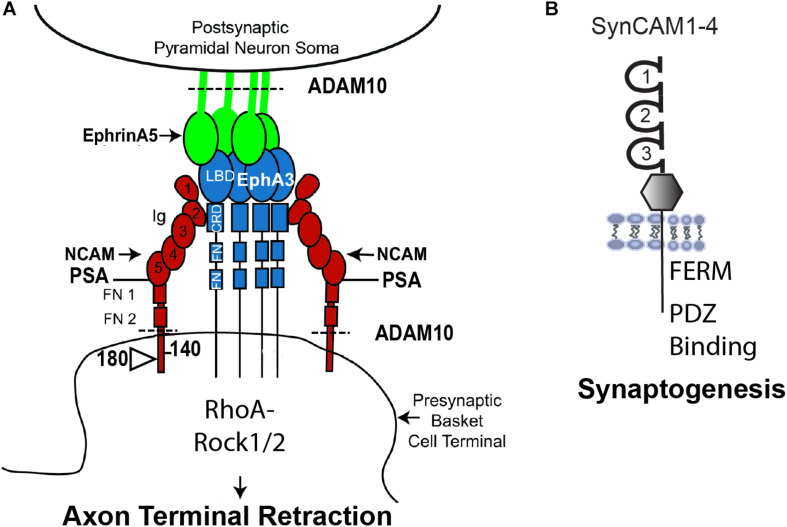
Model of NCAM Mediated Remodeling of Inhibitory Synapses**. (A)** NCAM1 (there is also a lesser studied NCAM2) contains 5 Ig and 2 FNIII domains, and alternative splicing produces 3 major isoforms with different sizes of the cytoplasmic domain. NCAM140 has the smallest cytoplasmic tail, while NCAM180 kDa has an insert in this sequence. NCAM 120 (not shown) is linked to the plasma membrane by a glycophosphatidyl inositol (GPI) tag and is expressed in glia. NCAM Ig1 and Ig2 domains mediate homophilic adhesion in both *cis* and *trans*. The extracellular region of all NCAM isoforms can be modified by polysialylation (PSA) on Ig5, which reduces homophilic binding affinity and promotes neural plasticity. NCAM engages the EphrinA receptor tyrosine kinase EphA3 through interaction of NCAM Ig2 with the cysteine-rich domain (CRD) of EphA3. GPI-linked EphrinA5 on pyramidal cell soma binds the ligand binding domain (LBD) of EphA3 and promotes clustering of PSA-NCAM and EphA3 on axon terminals of parvalbumin-expressing basket interneurons. PSA-NCAM, EphA3, and ADAM10 metalloprotease coordinate to regulate EphrinA/EphA-induced retraction of axon terminals of interneurons from the perisomatic region of cortical pyramidal neurons by downstream signaling through RhoA and Rho kinase (Rock1/2). ADAM10 cleaves PSA-NCAM and EphrinA5 (dotted line) to facilitate retraction. **(B)** SynCAMs1–4 are synaptic adhesion molecules with 3 Ig domains, an extracellular juxta-membrane region, and a conserved cytoplasmic tail that binds. FERM- and PDZ-containing proteins. SynCAMs engage in heterophilic interactions within the family, as well as *cis* and *trans* homophilic adhesion. SynCAM1 promotes synaptogenesis of excitatory synapses as well as synaptic maintenance.

A recently identified function for NCAM is in reducing the potential for inhibitory neurotransmission by pruning excess perisomatic inputs from basket interneurons during postnatal development of the prefrontal cortex (Figure 2A). This remodeling occurs in response to repellent EphrinA ligands (EphrinA2, A3, and A5), which bind the EphA3 receptor kinase in complex with the 140 kDa isoform of NCAM (Pillai-Nair et al., 2005; Brennaman et al., 2013; Sullivan et al., 2016, 2018, 2020). Loss of NCAM, EphA3, or EphrinA2/3/5 in mice increases the density of perisomatic inhibitory boutons on pyramidal neurons in layer 2/3 of the prefrontal cortex (Brennaman et al., 2013). EphrinA5, a glycophosphatidyl inositol (GPI)-linked protein, stimulates co-clustering of NCAM and EphA3 on basket cell terminals, which triggers retraction of the axon terminal by activating RhoA and its principal downstream effector Rho-associated protein kinase (ROCK1/2) (Brennaman et al., 2013; Sullivan et al., 2016). An integral component of the mechanism, ADAM10 (a disintegrin and metalloprotease-10) cleaves surface-bound NCAM and EphrinA5 to promote detachment and enable EphrinA-induced retraction (Figure 2A, dotted lines) (Brennaman et al., 2014). Laser scanning photostimulation (LSPS) of GABAergic interneurons expressing light-activated channel-rhodopsin in NCAM-deficient brain slices was shown to increase the inhibitory field of these neurons in the prefrontal cortex, consistent with an increase in the number of functional perisomatic synapses (Zhang et al., 2017). Conditional deletion of NCAM in PV-expressing interneurons in mice was recently demonstrated to constrain the extent of postnatal remodeling of perisomatic inhibitory synapses in the prefrontal cortex of homozygous PV-Cre:NCAM^flox/flox^:tdTomato mice (Sullivan et al., 2020). This study used live imaging in brain slices to reveal that PV+ perisomatic boutons are highly dynamic in wild type prefrontal cortex but much less dynamic in NCAM-deficient cortex. The PV-Cre: NCAM conditional mutant mice also exhibited impaired inhibitory neurotransmission, working memory, and social behavior (Sullivan et al., 2020).

Polysialylation (PSA) of NCAM on the Ig5 domain is a developmental modification that has a prominent influence on plasticity in cellular responses, including neuronal migration (Schuster et al., 2020) and modulation of extrasynaptic NMDA receptors (Sytnyk et al., 2017; Varbanov and Dityatev, 2017). Polysialylation of NCAM is achieved through the expression and activation of two poly-sialyltransferases, St8sia II, and St8sia IV (Hildebrandt et al., 2010). The 140 and 120 kDa NCAM isoforms promote the maturation of GABAergic basket cell synaptic fields in the visual cortex (Chattopadhyaya et al., 2013). PSA-modification of NCAM prevents the premature closure of the critical period of visual plasticity by restricting innervation of pyramidal cell soma by basket cell terminals, while non-PSA modified NCAM promotes synaptic targeting of these inputs (Di Cristo et al., 2007). In accord with the dynamic nature of basket cell synaptic puncta in the prefrontal cortex, inhibitory synapses in the visual cortex undergo pronounced GABA-dependent remodeling (Fu et al., 2012; Wu et al., 2012). Evidence suggests that a GABA activity-dependent “punishing signal” may be produced to eliminate inactive basket cell synaptic contacts (Baho and Di Cristo, 2012; Wu et al., 2012). It is interesting to speculate that EphrinA5 might serve as such a signal, as it is upregulated on the neuronal surface in an activity-dependent manner in neuronal cultures (Sullivan et al., 2020). In addition, PSA-NCAM is regulated by activity *in vivo* (Di Cristo et al., 2007). A plausible scenario is that active inhibitory synapses may prune weaker neighbors through EphrinA/NCAM/EphA3 repellent signaling to fine tune microcircuits during postnatal maturation (Figure 2A). Similarly, local competition has been shown to be involved in homeostatic regulation of excitatory synapses in both developing and mature brain (Turrigiano, 2012; Bian et al., 2015; Kehayas and Holtmaat, 2015; Oh et al., 2015; Stein and Zito, 2019).

There is accumulating evidence for developmental refinement of inhibitory connections in other brain regions. The density of GABA-positive boutons decreases from late adolescence to young adulthood in the auditory cortex (Moyer et al., 2015). Inhibitory terminals from the medial nucleus of the trapezoid body onto the lateral superior olivary nucleus are also eliminated upon maturation (Kotak and Sanes, 2014). Intrinsic interneurons in the thalamic dorsal lateral geniculate nucleus were found to remodel their arbors in response to retinal activity (Charalambakis et al., 2019). In the primary somatosensory cortex (S1), GABAergic inputs from the thalamus transiently integrate into cortical circuits and are refined postnatally, potentially serving to suppress excitability from emerging pyramidal cell connections (Marques-Smith et al., 2016). Inhibitory inputs from local interneurons onto pyramidal cells in S1 also undergo postnatal refinement (Cocas et al., 2016). These effects might be region specific, for example the connection probability of PV+ interneurons in S1 layer 2/3 shows only an insignificant decrease with developmental age (Packer and Yuste, 2011).

SynCAMs (1–4) represent a distinct class of Ig-family adhesion molecules that contribute to synapse formation and stability (Figure 2B). SynCAM1 participates in formation and stabilization of new synapses through complex homophilic and heterophilic binding interactions (Frei and Stoeckli, 2017). SynCAM1 and SynCAM2 form a trans-synaptic complex that promotes formation and maintenance of excitatory synapses (Fogel et al., 2007). These SynCAM interactions are mediated by site-specific *N*-glycosylation on the extracellular binding interface. Specifically, the *N*-glycans in SynCAM2 Ig1 decrease adhesion, while glycosylation in SynCAM2 Ig1 increases adhesion (Fogel et al., 2010). The cytoplasmic domain of SynCAM1 contributes to synapse stabilization by recruiting FERM- and PDZ-containing scaffold molecules (Figure 2B; Frei and Stoeckli, 2017). SynCAM1 also facilitates spine maturation and stability shown by increased spine density and size in SynCAM1 overexpressing mice (Robbins et al., 2010), although contrasting findings were noted in SynCAM1 null mice (Korber and Stein, 2016). Dentate gyrus neurons from SynCAM1 overexpressing mice also exhibit increased spine density and size, as well as increased excitatory transmission manifested by elevated mEPSC frequency and amplitude (Doengi et al., 2016). Furthermore, SynCAM1 null mice display delayed maturation of the visual cortex, the presence of immature cortical PV+ interneurons, and decreased thalamacortical inputs onto PV+ interneurons (Ribic et al., 2019). Recent studies suggest that SynCAM1 may interact with other Ig-class adhesion molecules such as L1-CAMs and NCAM to mediate synaptic stability, as indicated by proteome mapping of the synaptic cleft in cultured cortical neurons (Cijsouw et al., 2018).

## Role of Ankyrin Interaction With Neural Adhesion Molecules in Synaptic Stabilization

Synaptic stabilization is a postnatal mechanism to regulate synapse density by strengthening and maintaining synapses. Though persistence and elimination of spines have been extensively studied, recent research has focused on mechanisms of synaptic structural stability (Meyer et al., 2014). Synaptic stabilization involves the clustering and complexing of synaptic adhesion molecules, which enhances the physical interaction of pre- and post-synaptic membranes. Additional interactions between the L1 family members NrCAM and CHL1 stabilize synapses on GABAergic inhibitory neurons by complexing with sensory derived proteins NB2 (Contactin5) of the Ig superfamily and contactin-associated protein Caspr4 (Ashrafi et al., 2014). CHL1 expression on stellate cells in the cerebellum may also stabilize synapses through heterophilic interactions between Purkinje dendrites or homophilic interactions with Bergman glia in postnatal mice (Ango et al., 2008).

**FIGURE 3:**
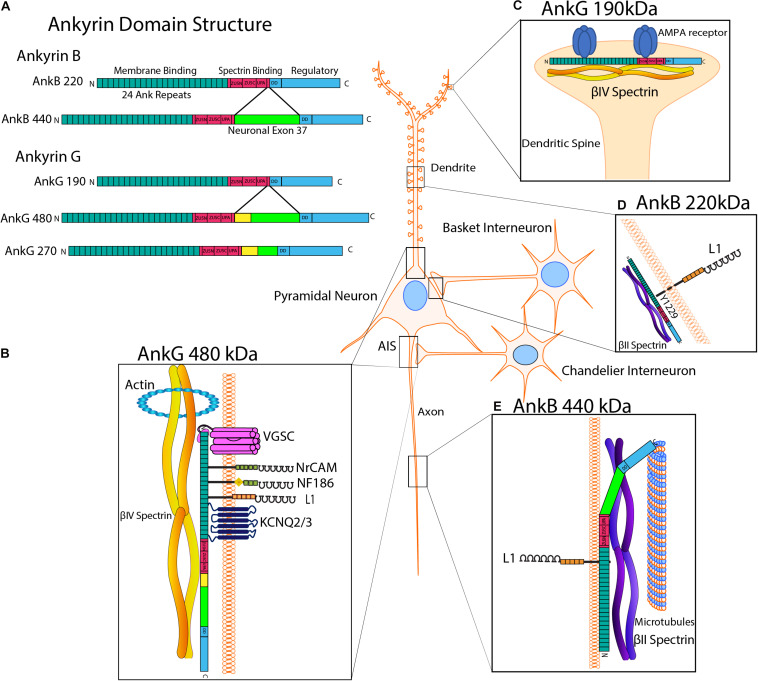
Ankyrin Structure and Subcellular Localization with L1 Interactions**. (A)** Human Ankyrin B and Ankyrin G isoforms. Ankyrin is comprised of an N-terminal membrane binding domain consisting of 24 Ank repeats that recruit L1-CAMs and ion channels, a spectrin binding domain containing ZU5-N, ZU5-C and UPA subdomains, a death domain (DD), and a C-terminal regulatory tail. Ankyrin B has two major splice variants, isoforms 220 kDa and giant AnkB 440 kDa, which has an insert encoded by neuronal exon 37 (bright green). Ankyrin G splice variants include isoforms of 190, 270, and 480 kDa, the latter two of which have inserts encoded by neuronal partial exon 37 and giant exon 37, respectively. AnkG 270 and 480 also contain a serine-rich domain (yellow), which mediates targeting to the axon initial segment (AIS). **(B)** AnkG 480 kDa. At the AIS, giant AnkG 480 kDa clusters and tethers proteins, including voltage gated sodium channels (VGSCs), KCNQ2/3 potassium channels, NrCAM, NF186 and L1, to the actin-spectrin cytoskeleton. The AnkG spectrin binding domain binds βIV-spectrin, which links to actin at periodic ring structures. AnkG 480 is also localized to the somato-dendritic compartment of pyramidal neurons. **(C)** AnkG 190 kDa. AnkG 190 is present in dendritic spines, where it stabilizes AMPA receptors by promoting the formation of complexes of AnkG, AMPA receptors and βIV-spectrin at synapses. **(D)** AnkB 220 kDa. AnkB is reversibly recruited to the cytoplasmic domain of L1 on pyramidal cell soma at PV+ GABAergic interneuron synapses. This binding is fostered by dephosphorylation of L1 at Tyr1229, whereas phosphorylation of this residue results in loss of Ankyrin binding. AnkB220 is also localized to pyramidal cell dendrites and soma. **(E)** AnkB 440 kDa. Giant AnkB (440 kDa) binds to both L1 and microtubules in axons. AnkB 440 kDa bundles microtubules and tethers them to the actin-βII spectrin cytoskeleton, which helps maintain structural stability of the axon.

One mechanism by which stability of newly formed synapses is achieved is through the recruitment of scaffolding proteins such as the actin cytoskeletal adaptors Ankyrin B/G and their binding partner β-spectrin to the cytoplasmic domains of L1-CAMS (Figure 3; Jenkins and Bennett, 2001; Doengi et al., 2016; Yang et al., 2019). Reversible binding of the L1-CAM cytoplasmic domain to Ankyrin is regulated by dephosphorylation of a tyrosine residue (Y1229) in a conserved motif FIGQ/AY (Figure 3D; Garver et al., 1997; Yamasaki et al., 1997; Needham et al., 2001; Guan and Maness, 2010). Mutation of Tyr1229 in L1 to histidine occurs in the L1 syndrome of intellectual disability (Hortsch et al., 2014). A mouse model of this disorder harbors an L1 knock-in point mutation (Tyr1229His) that impairs L1-Ankyrin binding (Buhusi et al., 2008). These “L1YH” mice exhibit decreased synapse density of GABAergic interneurons targeting pyramidal cell soma in the prefrontal cortex (Guan and Maness, 2010). L1YH mice also lose the L1-AnkG-β4-spectrin interaction at the AIS (Tai et al., 2019), and L1 null mice exhibit reduced numbers of hippocampal perisomatic inhibitory synapses (Saghatelyan et al., 2004). L1 mutant mice that harbor mutations in nuclear receptor binding motifs show decreased inhibitory GAD67+ and excitatory vGlut+ synaptic puncta on cerebellar Purkinje cells (Kraus et al., 2018). Ankyrin links other membrane and scaffold proteins to the cytoskeleton to further achieve synaptic stability.

Multiple alternative splice variants of Ankyrin B (AnkB) are encoded by the ANK2 gene (Figure 3A). The AnkB 220 kDa isoform is ubiquitously expressed, and in pyramidal neurons appears to localize to dendritic and soma subcellular compartments (Figure 3D; Kunimoto, 1995). Giant AnkB (440 kDa) has a large neuronal-specific insert encoded by exon 37 and associates with the axonal plasma membrane through L1 engagement (Figure 3E; Yang et al., 2019). Giant AnkB binds and bundles microtubules (MTs) *via* a 12-residue motif unique to this isoform, tethering MTs to the actin cytoskeleton (Figure 3E). This interaction may maintain the structural stability and fasciculation of MTs in axons (Chen et al., 2020). Giant AnkB mutant mice and L1YH mice show transiently increased axon branching, which is likely mediated through MT growth (Yang et al., 2019). Additionally, neuromuscular junctions in *Drosophila* mutants lacking the giant AnkB isoform (Ank2-L) exhibit increased synaptic retractions and fragmented presynaptic membranes (Weber et al., 2019).

Ankyrin G, encoded by the ANK3 gene, mediates recruitment, coordination, and clustering of multiple proteins at the AIS: voltage gated sodium channels (VGSCs), KCNQ 2/3 potassium channels, Neurofascin 186 (NF 186), and β4-spectrin (Figures 3A,B; Jenkins et al., 2015). The AIS, located between neuronal somatodendritic and axon domains, is responsible for the initiation of action potentials by maintaining ion channels and regulation of cargo transport and neuronal polarity (Kole et al., 2008; Rasband, 2010; Leterrier, 2018). Securing these hetero-complexes to the actin/spectrin cytoskeleton allows for proper action potential initiation, excitability regulation and maintenance of distinct axon and somatodendritic compartments (Gulledge and Bravo, 2016; Lazarov et al., 2018; Yang et al., 2019; Goethals and Brette, 2020). The giant 480 kDa AnkG isoform is needed for development and function of the AIS. Knockdown of 480 kDa AnkG in cultured hippocampal neurons diminishes clustering of β4-spectrin, VGSCs and NF186 (Jenkins et al., 2015). In exon 37 null mice with a specific deletion of 480kD AnkG, recruitment of AnkG to the AIS and clustering of β4-spectrin, NF186, VGSCs, and KCNQ2 were lost in Purkinje neurons (Jenkins et al., 2015). AnkG 480kD also mediates the stability of GABAergic synapses *via* interaction with GABA_A_ receptor-associated protein, which inhibits endocytosis in hippocampal neurons (Tseng et al., 2015). AnkG 480 deletion results in GABAergic synapse loss in the hippocampal CA1 region and the cerebral cortex postnatally (Tseng et al., 2015). In cortical neuron cultures, it was found that AnkG helps to stabilize AMPA receptors at synapses by promoting the formation of multiprotein complexes consisting of AnkG, AMPA receptors, and β-spectrin (Figure 3C; Smith et al., 2014). Additional studies on human AnkG in psychiatric disorders have been done (Luoni et al., 2016) and AnkG has been recently reviewed in Salzer (2019).

The association of L1-CAM Neurofascin 186 (NF186) with AnkG is required for formation and stabilization of pinceau synapses in the cerebellar Purkinje neurons (Ango et al., 2004). A NF186 gradient directs basket interneuron axons to the AIS of Purkinje cells during pinceau synapse formation. In Purkinje neurons lacking AnkG, NF186 is uniformly distributed, and basket cell axons lack directional growth toward the AIS during postnatal maturation (Ango et al., 2004). The AnkG 190 kDa isoform is localized to nanodomains where it contributes to spine morphology (Smith et al., 2014). β-spectrin is needed to target AnkG to dendritic spines in cortical neurons of adult mice. Mutation of the AnkG 190 kDa isoform in cortical neurons prevents binding of β-spectrin and targeting of AnkG to spines. AnkG targeting of spines is critical for AMPA receptor clustering and regulation (Smith et al., 2014).

## Perineuronal Net Proteins Stabilize Synapses, Bind Neural Adhesion Molecules, and Regulate Synaptic Plasticity

**FIGURE 4:**
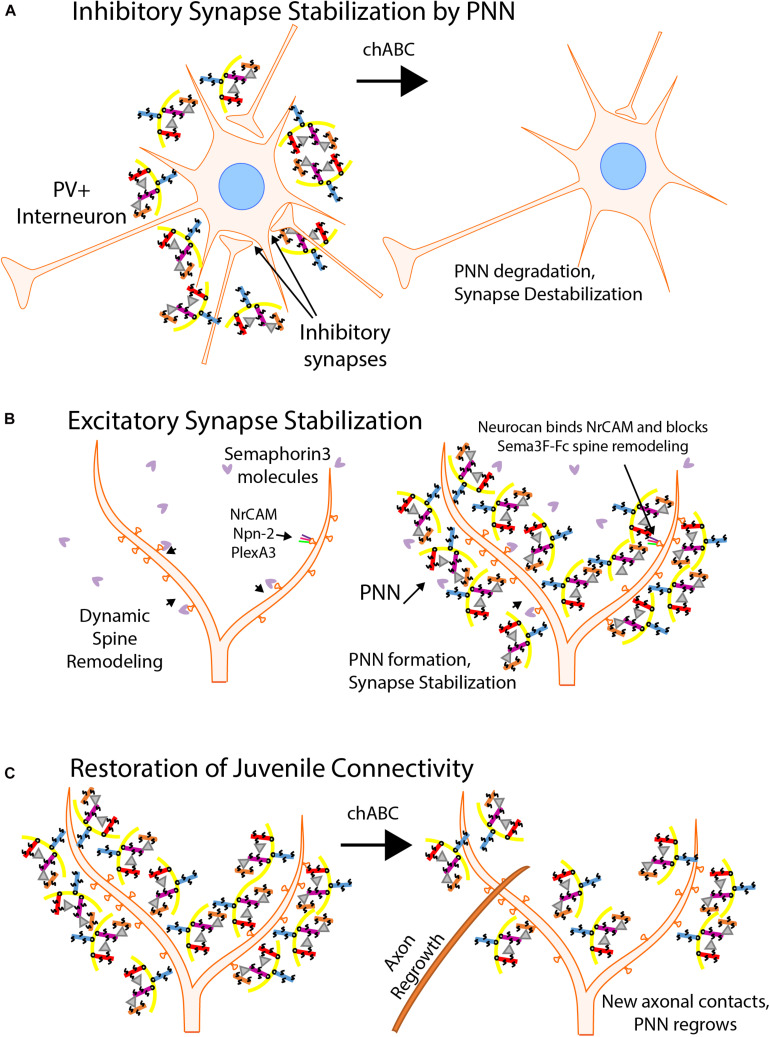
Perineuronal Nets Regulate Synaptic Stability. **(A)** Inhibitory synapses are stabilized by Perineuronal Nets (PNNs). The PNN matrix consists of chondroitin sulfate proteoglycans (CSPGs) (aggrecan, purple; neurocan, red; brevican, orange; versican, blue), hyaluronic acid (yellow), tenascin-R (gray triangles), and linker proteins (not shown). PNNs surround soma of maturing inhibitory interneurons, particularly PV+ basket cells, and stabilize perisomatic synaptic connections. Chondroitinase ABC degradation (chABC) of the CSPGs cause the degradation of the PNN and decreases inhibitory synapse strength and number. **(B)** PNNs inhibit dendritic spine remodeling and stabilize excitatory synapses. PNNs develop around neuronal soma and dendrites as a protective mesh as synaptic maturation occurs, decreasing neuronal plasticity. Maturing neurons are protected from spine pruning in response to class 3 Semaphorins by interaction of the CSPG neurocan with NrCAM in the Sema3F holoreceptor complex. **(C)** Juvenile neuronal connectivity following chABC treatment. Treatment of mature neuronal cells with chABC degrades the PNN and in some neuronal regions restores juvenile connectivity by allowing new axonal connections to be formed. Normally, CNS injuries prohibit reconnection of synapses due to the forming PNN. Following chABC treatment, axonal reconnections can be made and PNNs regrow over a few weeks, stabilizing the synapses.

Extracellular proteins that contribute to synapse stabilization constitute the perineuronal net (PNN) (Figure 4). PNNs contain multiple molecules including chondroitin sulfate proteoglycans (CSPGs) with glycosaminoglycan (GAG) side chains, hyaluronic acid, tenascin-R, and linker proteins, which combine to form a mesh-like structure that surrounds maturing neurons, particularly at the soma of PV+ basket cells (Friedlander et al., 1994; Koppe et al., 1997). The PNN develops postnatally at approximately the same time that synapses are formed and stabilized. Neuronal plasticity decreases as the PNN arises and binds to neuronal surface proteins, stabilizing synaptic contacts (Pizzorusso et al., 2002; Wang and Fawcett, 2012; Carulli et al., 2016). Interestingly, PV+ interneurons that are surrounded by PNNs are more mature than interneurons without PNNs (Carceller et al., 2020), potentially providing greater stability to established perisomatic synapses. Additionally, examining cells following enzymatic digestion of PNNs by *Proteus vulgaris* chondroitinase ABC (chABC) revealed decreases in the number and stability of perisomatic synaptic puncta in mice (Figure 4A). chABC treatment of tenascin-R null mice also showed reduced perisomatic inhibition (Bukalo et al., 2001). Similar results were seen in normal rats (Lensjo et al., 2017).

There are five principal CSPGs expressed in the brain: aggrecan, versican, phosphacan, brevican, and neurocan. Except for aggrecan, CSPGs are expressed in discrete regions of the brain. PNNs formed during maturation restrict plasticity as shown in the visual cortex (Pizzorusso et al., 2002), amygdala (Gogolla et al., 2009), prefrontal cortex (Slaker et al., 2015), and hippocampus (Geissler et al., 2013). Strikingly, digestion of the PNN with chABC re-establishes juvenile-like synaptic remodeling in the amygdala (Gogolla et al., 2009), auditory cortex (Happel et al., 2014), perirhinal cortex (Romberg et al., 2013), and spinal cord (Harris et al., 2010). Studies have begun to elucidate the role of PNNs in adolescent development as well as their role in disease states such as schizophrenia, bipolar disorder, dyslexia, and autism (Pantazopoulos and Berretta, 2016; Sorg et al., 2016). For example, post-mortem examination of schizophrenic brains showed a marked decrease in PNNs by *Wisteria Floribunda* agglutinin histochemistry (Mauney et al., 2013).

Neurocan is a prominent organizer of the PNN and one of the first CSPGs to be expressed in the maturing neocortex. Early research on neurocan showed its prevalence in the brain (>20% of the soluble CSPGs) and its ability to limit neurite outgrowth in culture (Friedlander et al., 1994). More recent studies have shown that neurocan can effectively block synaptogenesis (Celio and Blumcke, 1994; Celio et al., 1998; Dityatev and Schachner, 2003; McRae et al., 2010; Soleman et al., 2013; Suttkus et al., 2016; Sullivan et al., 2018). During postnatal development, neurocan assembles around processes, dendritic spines, and soma of pyramidal neurons. Neurocan has been shown to inhibit Sema3F-mediated spine retraction in cortical neurons in culture by binding NrCAM and inhibiting its function within the Sema3F holoreceptor (Figure 4B; Mohan et al., 2018). Neurocan also binds to the Ig2 domain of NCAM at the EphA3 binding site, inhibiting the repellant response to EphrinA5 exhibited by remodeling basket cell terminals (Sullivan et al., 2018).

Another prominent CSPG expressed in brain is brevican. Like neurocan, brevican is involved in synaptic plasticity. In mouse studies examining learning and memory, brevican levels decreased at the beginning of the learning phase and increased after learning was established (Niekisch et al., 2019). Another study found that brevican was essential for the maturation of inputs from excitatory neurons onto PV+ interneuron soma (Favuzzi et al., 2017). Brevican deletion or knockdown in PV+ interneurons causes deficits in spatial working memory and short-term memory, but not long-term memory. Additionally, brevican null mutant mice show longer action potential transmission delays (Blosa et al., 2015). In brevican null mice, PNNs appear intact suggesting that the primary role of this CSPG may be to establish and maintain synaptic function.

## Role of CSPG in Recovery After CNS Injury

A promising future direction of research into PNN function is CNS injury and repair. Using chABC and its ability to restore juvenile connectivity conditions in treated tissue, the enzyme has been employed as a possible therapeutic option for CNS injuries in animal models (Figure 4C). Following injury to the CNS, a scar forms that consists of glial cell infiltration and an increase in PNN molecules, including CSPGs. This scar grows more quickly than recovery of injured neurons, and blocks neurite growth (Griffin and Bradke, 2020). Digestion with chABC may be an effective treatment for the restoration of neuronal connectivity after such injury. After an acute cervical dorsal column crush lesion, adult rats were able to regenerate a limited number of sensory and cortical spinal tract motor axons and restore both sensory and motor control over limbs affected by the injury after treatment with chABC (Bradbury et al., 2002). Another study showed pericontusional axon sprouting increases in chABC treated rats after a controlled cortical impact injury (Harris et al., 2010). Neurocan has been shown to inhibit the growth of axons in a brain injury model and, along with other CSPGs, is upregulated in injured brains (Asher et al., 2000). Upregulation can be a response to increased levels of transforming growth factor beta (TGF-β) and epidermal growth factor. Recent advances in the deployment of the chABC enzyme show promise. By targeting chABC to axons, neurite extension in SH-SY5Y neurons was significantly increased, even over cells that were transfected with a non-targeted variant of the enzyme (Day et al., 2020). Despite the successes in animal models, no clinical trials using the enzyme have been reported. Innovations with enzyme presentation and continued research with PNNs may allow for a reliable and effective option to be used to restore neuronal connectivity in patients with CNS injuries.

## Discussion

The synapse is regulated, strengthened, established, and removed through a host of molecules orchestrated to achieve an optimal functional level of connectivity among excitatory and inhibitory synapses. Synaptic disorders including autism spectrum disorders, schizophrenia, Fragile X syndrome, or intellectual disability often feature disruptions in the function of synapse regulating proteins such as Ig-CAMs, Ankyrins, or CSPGs. The L1 family of Ig-class CAMs are important as obligate subunits of Semaphorin-3 holoreceptor complexes, which mediate developmental spine pruning. All L1-CAMs recruit the spectrin-actin adaptor protein Ankyrin, a high confidence autism risk factor that is critical for both synaptic remodeling and stabilization. At inhibitory synapses, EphrinA ligands and EphA receptor tyrosine kinases function through binding to neural adhesion molecule NCAM to limit the number of perisomatic synapses of basket interneurons, important for both working memory and sociability. The PNN also participates in regulating synaptic plasticity in the developing brain. As the matrix is built, axonal contacts to both dendritic spines and perisomatic regions of pyramidal cells and interneurons are reinforced, plasticity characteristic of juvenile neurons decreases, and new opportunities for connections are blocked. By considering all of these molecular mechanisms as a comprehensive system, new therapeutic targets meant to ameliorate cognitive dysfunction may become available.

## Author Contributions

BD organized the manuscript. All authors contributed to the writing.

## Conflict of Interest

The authors declare that the research was conducted in the absence of any commercial or financial relationships that could be construed as a potential conflict of interest.
